# Supplementary material for: Ultra-short pulse laser acceleration of protons to 80 MeV from cryogenic hydrogen jets tailored to near-critical density
Source: Nat Commun. 2023 Jul 7;14:4009. doi: 10.1038/s41467-023-39739-0 (PMC10329016; doi:10.1038/s41467-023-39739-0)
Supplement: Supplementary file 1 — Supplementary Information [file 41467_2023_39739_MOESM1_ESM.pdf]

Supplementary Information

Ultra-short pulse laser acceleration of protons to 80 MeV from cryogenic hydrogen jets tailored to near-critical density

Martin Rehwald,[1,2,*] Stefan Assenbaum,[1,2] Constantin Bernert,[1,2] Florian-Emanuel Brack,[1,2] Michael Bussmann,[1,3] Thomas E. Cowan,[1,2] Chandra B. Curry,[4,5] Frederico Fiuza,[4] Marco Garten,[1,6] Lennart Gaus,[1,2] Maxence Gauthier,[4] Sebastian Göde,[7] Ilja Göthel,[1,2] Siegfried H. Glenzer,[4] Lingen G. Huang,[1] Axel Huebl,[1,6] Jongjin B. Kim,[4] Thomas Kluge,[1] Stephan Kraft,[1] Florian Kroll,[1] Josefine Metzkes-Ng,[1] Thomas Miethlinger,[1,2] Markus Loeser,[1] Lieselotte Obst-Huebl,[1,6] Marvin Reimold,[1,2] Hans-Peter Schlenvoigt,[1] Christopher Schoenwaelder,[4,8] Ulrich Schramm,[1,2] Mathias Siebold,[1] Franziska Treffert,[4,9] Long Yang,[1] Tim Ziegler,[1,2] and Karl Zeil[1]

[1] *Helmholtz-Zentrum Dresden - Rossendorf, Institute of Radiation Physics, Bautzner Landstr. 400, 01328 Dresden, Germany*
[2] *Technische Universität Dresden, 01062 Dresden, Germany*
[3] *Center for Advanced Systems Understanding (CASUS), 02826 Görlitz, Germany*
[4] *High Energy Density Science Division, SLAC National Accelerator Laboratory, Menlo Park, California 94025, USA*
[5] *University of Alberta, Edmonton, Alberta T6G 1H9, Canada*
[6] *Present address: Lawrence Berkeley National Laboratory, Berkeley, California, 94720, USA*
[7] *European XFEL GmbH, Holzkoppel 4, 22869 Schenefeld, Germany*
[8] *Friedrich-Alexander Universität Erlangen-Nürnberg, 91054 Erlangen, Germany*
[9] *Technische Universität Darmstadt, 64289 Darmstadt, Germany*

[*] m.rehwald@hzdr.de

SUPPLEMENTARY DISCUSSION: ANGULAR PROTON EMISSION DISTRIBUTION FROM THE DEPTH DOSE DETECTOR

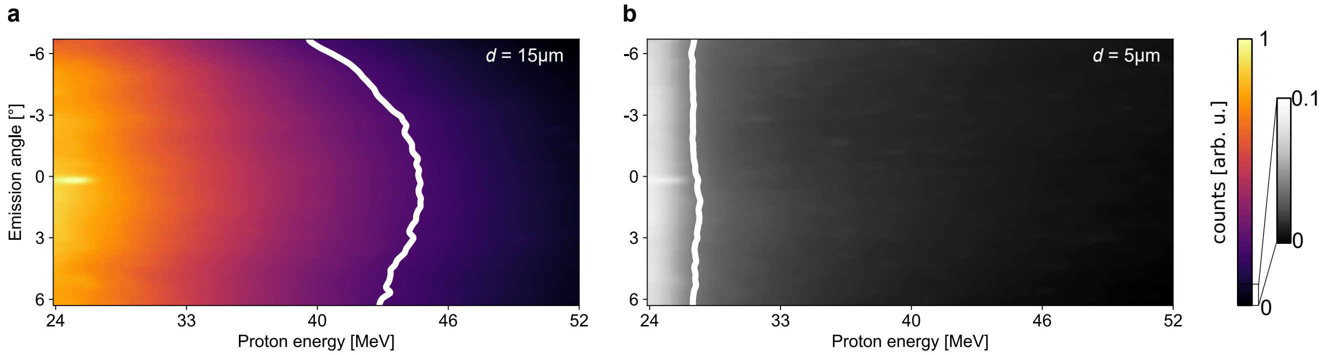

Supplementary Fig. 1. **Proton profile measurements using the depth dose detector.** Horizontal cross-sections of the angular proton depth dose profile recorded using an on-shot scintillator based depth dose detector (DDD) along the laser propagation direction. **a** is plotted for an expanded target ($d = 15\,\mu m$) yielding a forward peaked proton distribution as indicated by the white contour line and compared to the unexpanded case **b** featuring only a slight profile curvature. Note that the recorded signal strength is ten times smaller in the unexpanded case.

The change in the proton emission distribution inferred from the TPS measurements in Fig. 3 of the main text is qualitatively supported by the recordings of the on-shot depth dose detector (DDD). This plastic scintillator-based detector records the angularly resolved proton depth dose profile within the horizontal plane behind the target. It covered an opening angle of about $\pm 7°$ with respect to the laser propagation direction and an additional energy filter in front of the 2 mm thick scintillator plate set the low energy detection cut-off to 24 MeV. Note that the scintillator-based detector is also sensitive to high-energy electrons and x-rays from the high-intensity laser plasma interaction, although their absorption is smaller than that of protons. While this background complicates the detection of maximum proton energies, we found that the dependence of the depth dose distribution on the emission angle can be well distinguished. DDD recordings of a shot on an expanded ($d = 15\,\mu m$) and an unexpanded hydrogen jet ($d = 5\,\mu m$) are shown in Supplementary Fig. 1a and b, respectively. While a slight profile curvature is found for the unexpanded case, the depth dose profile obtained for the pre-expanded configuration shows a clear dependence on the emission angle as indicated by the white contour line. This agrees well with the shift of the proton emission direction towards the laser forward direction for increased shadow diameters as inferred from the TPS measurements.

SUPPLEMENTARY DISCUSSION: PIC SIMULATIONS WITH VARYING OFFSET OF THE LASER WITH RESPECT TO THE TARGET AXIS

A series of PIC simulations was performed to study the influence of non-central hits for the three target expansion states discussed in the main text. For various lateral target position offsets between 0 µm and 5 µm, the proton energy spectra in the xy-plane as a function of the emission angle are shown in Supplementary Fig. 2, 3 and 4.
For the unexpanded target configuration in Supplementary Fig. 2, the maximum proton energies E_{max} are almost constant and independent of the emission angle for small offsets. This is due to the mostly isotropic proton emission perpendicular to the jet axis resulting from TNSA. Starting from position offsets of 1.5 µm, the most energetic protons are deflected toward progressively increasing angles reducing E_{max} in the laser forward direction. We now consider the best 1/3 of the laser shots (as in Fig. 3a in the main text) in order to compare E_{max} in laser forward direction from the simulations with the observed distribution of the proton energies in the experiment. For the measured target position jitter of 5 µm, these shots are expected to have a maximum offset of about 2 µm. We observe maximum proton energy fluctuations in laser forward direction between 20 MeV and 38 MeV in the experiment that are in good agreement with the 26 MeV (2 µm offset) to 35 MeV (0 µm offset) seen in the simulation. This demonstrate that the target position offset is the dominant contribution to the energy fluctuations in the experiment.

Similar good agreement between simulation and measurement is obtained for largely expanded targets. In the simulations for a target diameter of 33.5 µm (see Supplementary Fig. 3), we observe a low energetic component (about 10 MeV) emitted isotropically and an energetic proton beam (25 MeV) accelerated by MVA with a 20° divergence angle. With increasing target position offset MVA protons are deflected from the laser forward direction. However,

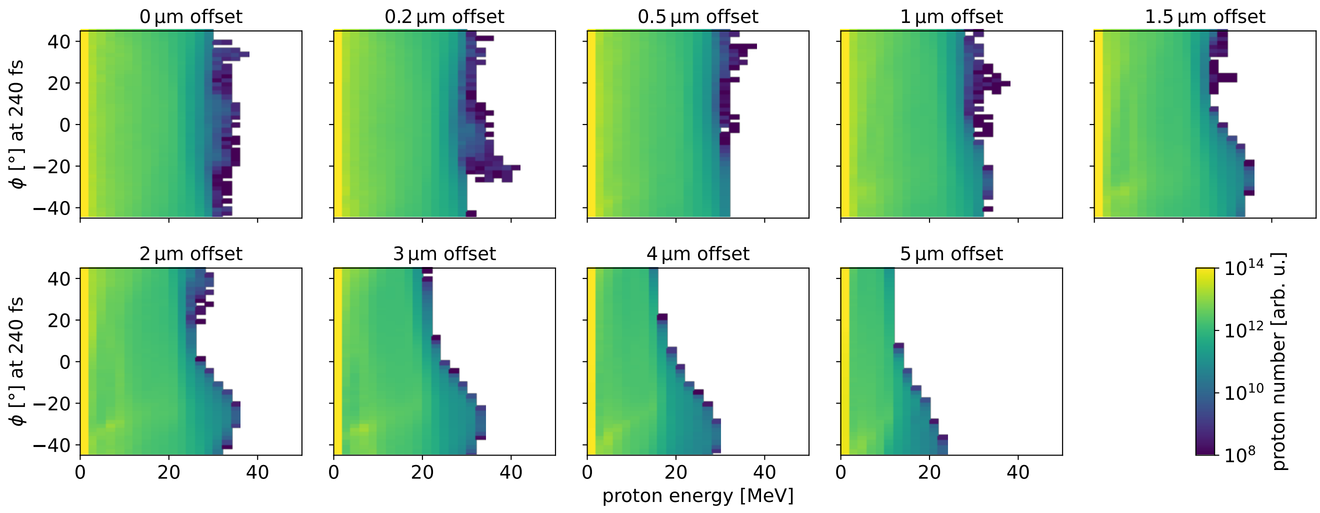

Supplementary Fig. 2. **Proton emission distributions in the unexpanded case for a scan of the laser target overlap.** PIC simulation results showing the calculated (at 240 fs) proton energy spectra in the xy-plane as a function of the emission angle ϕ for a target diameter of 6.2 μm and various offsets in the lateral target position between 0 μm and 5 μm.

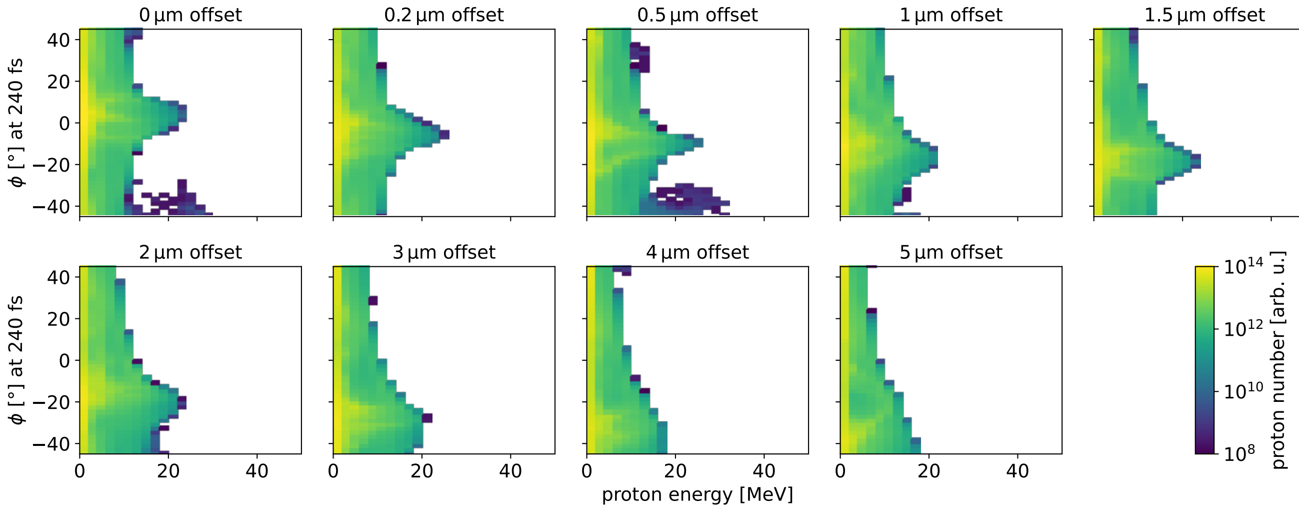

Supplementary Fig. 3. **Proton emission distributions in the largely expanded case for a scan of the laser target overlap.** PIC simulation results showing the calculated (at 240 fs) proton energy spectra in the xy-plane as a function of the emission angle ϕ for a target diameter of 33.5 μm and various offsets in the lateral target position between 0 μm and 5 μm.

the energies of the isotropically emitted component remain constant and leads to about 10 MeV even at the highest simulated offset of 5 μm. Given the limited amount of shots in the experiment with largely expanded targets (9 shots in total for d> 30 μm), the distribution of the observed maximum proton energies between 10 MeV and 20 MeV agrees well with the simulation results.

Lastly, simulation runs with non-central hits for the optimal target expansion are displayed in Supplementary Fig. 4. Here, the emission direction and the energy of the most energetic particles sensitively varies with the exact target position. As such, the energies in laser forward direction are reduced from 110 MeV at 0 μm offset to about 30 MeV at 2 μm offset. This large fluctuations in $E_{\max}$ reproduces the observation in the experiment where for the best 1/3 of the shots $E_{\max}$ was measured between 20 MeV and 80 MeV (see Fig. 3a in the main text). In summary, the simulations with non-central hits yield impressive match with the proton energy fluctuations observed in the experiments and

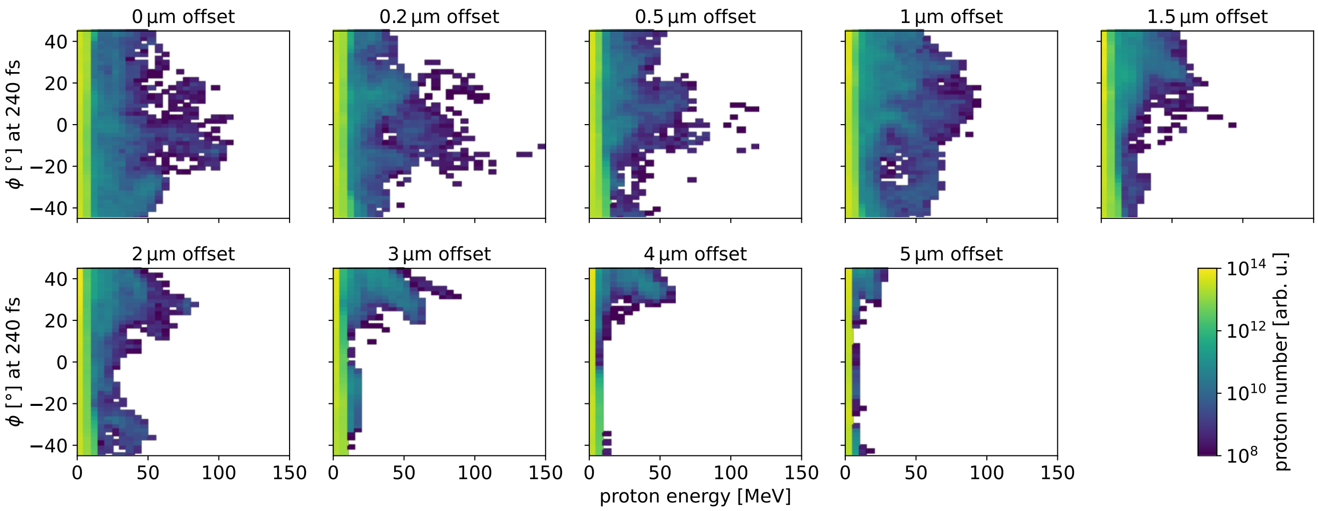

Supplementary Fig. 4. **Proton emission distributions in the optimal expansion case for a scan of the laser target overlap.** PIC simulation results showing the calculated (at 240 fs) proton energy spectra in the xy-plane as a function of the emission angle ϕ for a target diameter of 18.5 µm and various offsets in the lateral target position between 0 µm and 5 µm. Note the different energy scale with respect to Supplementary Fig. 2 and Supplementary Fig. 3.

show that these fluctuations are dominated by the spatial laser target overlap for all density regimes.

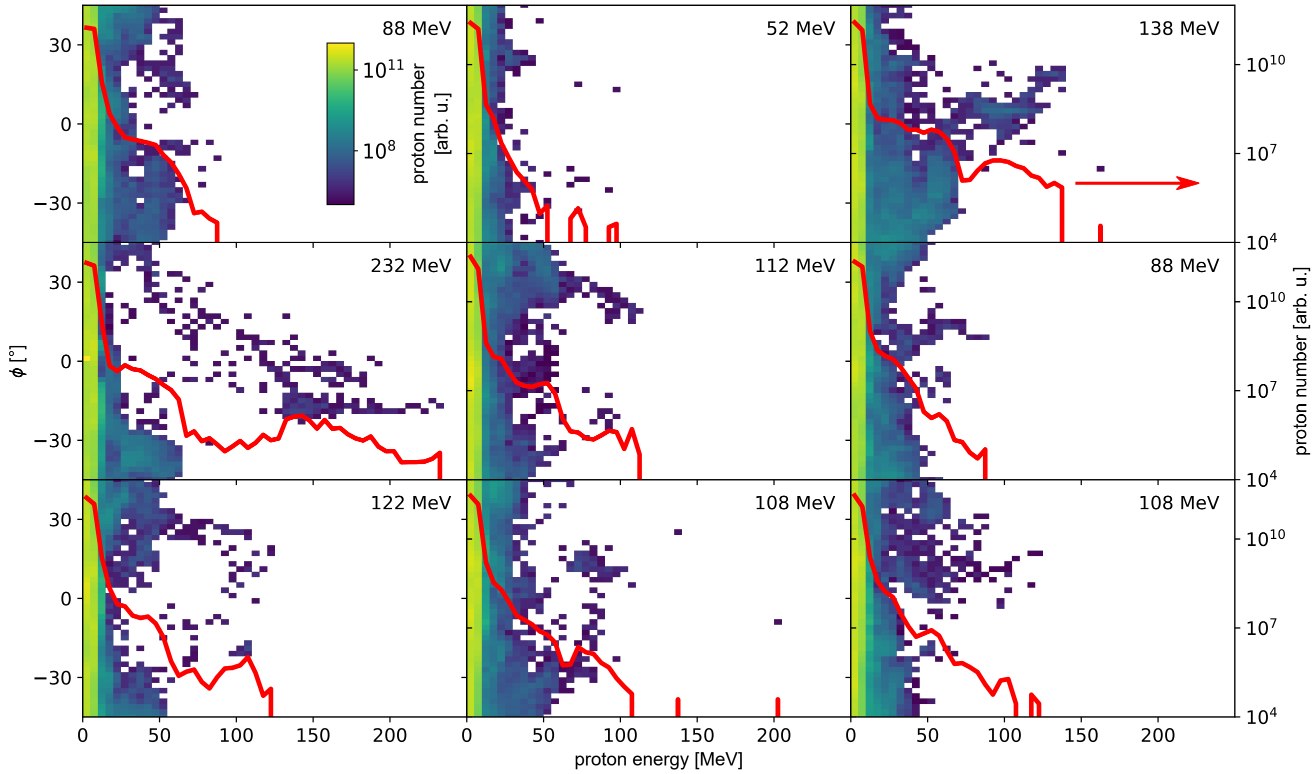

Supplementary Fig. 5. **Proton emission distribution from 9 simulation runs with the same configuration.** PIC Simulation results 240 fs after the peak pulse intensity reached the target center. The calculated proton energy spectra in the xy-plane as a function of emission angle ϕ are shown for 9 simulation runs with the same nominal laser and target (diameter 21.6 µm) configuration that yields the largest deviation between runs. $\phi = 0°$ denotes the laser propagation direction. Proton energy spectra averaged within the emission angle of $\pm 45°$ are shown by the red lines (right y-axis). The number in the upper right corner of each panel indicate the maximum proton energy taken.

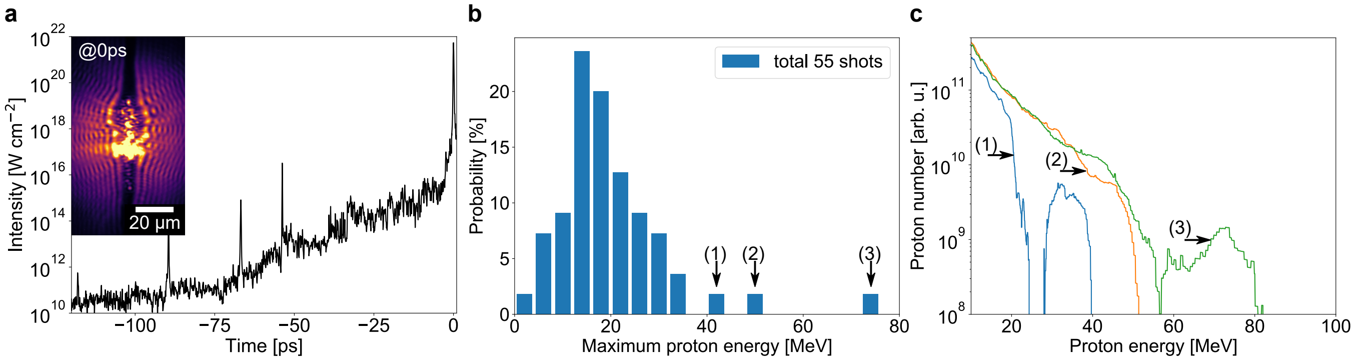

Supplementary Fig. 6. **Proton acceleration with intrinsic laser contrast conditions.** **a** Temporal intensity profile of the DRACO PW laser pulse without using the plasma mirror device. As a result of these intrinsic laser contrast conditions, the hydrogen jet has already expanded to a shadow diameter of about 15 μm at the time of peak intensity (see shadowgraphy image in **a**). The onset time at which the pre-expansion of the target sets in is a few tens of picoseconds before the arrival of the peak derived from the threshold intensity of dielectric breakdown [1]. The target density profile generated by the over tens of picoseconds accumulated laser light is expected to differ from the density distributions discussed in the main text and cannot be controlled independently as with the short-pulse pre-pulse or assessed by our probing technique (being sensitive only to the plasma scale length and not to longitudinal modulations of the bulk density). The distribution of the maximum proton energies (for a total of 55 shots with 23 J on target) in the laser forward direction using intrinsic laser contrast conditions is displayed in **b**. The large shot-to-shot fluctuation is again dominated by the varying spatial overlap of the laser focus spot and the hydrogen jet. While in the majority of the shots energies below 35 MeV are measured, three shots (labeled with (1)-(3)) with higher maximum proton energies are observed, the highest at almost 80 MeV. The measured proton energy spectra for these three shots are shown in **c**. Non-exponential proton spectra in two of the three shots are an additional indication for the target density profiles to differ from the short-pulse pre-pulse case. The single shot performance demonstrates that energies of 80 MeV are achievable without the use of the plasma mirror representing otherwise the main limitation of real repetition rate operation.

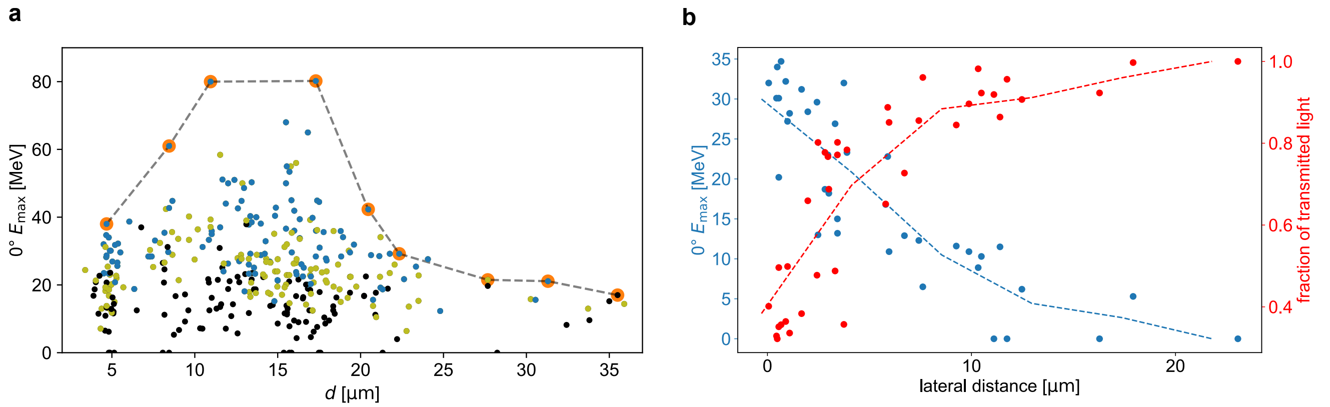

Supplementary Fig. 7. **Maximum proton energies in relation to the spatial overlap of laser focus and target position.** **a** extends the Fig. 3a of the main text, showing the entire scan (350 shots in total) of maximum proton energies measured in laser propagation direction as a function of the shadow diameter d. Different colors indicate different transmission cut-offs. The highest proton energies are measured for the 33% of shots with the least amount of light bypassing the target (blue dots as in Fig. 3a of the main text). Black and yellow dots represent each one-third of the shots with the largest and intermediate transmission, respectively. In particular for the black dots, the maximum proton energies are lower due to the insufficient spatial overlap of the jet and laser focus position. Best performing shots within 4 μm bins of the shadow diameter are furthermore highlighted by the orange circles. The comparatively low number of shots at the highest energy and their clear distinction from the overall distribution is due to the simultaneous requirement for perfect laser target overlap, ideal on-shot laser parameters and emission of the fastest protons in the detection direction of the TPS. The dominance of the lateral target position jitter as the main source of fluctuation in the maximum proton energies is shown for a subset (one day of experiments) with a shadow diameter of 5 μm in **b**. With increasing lateral distance from the central position, the proton energy (blue dots, left y-axis) is decreasing and the fraction of transmitted light (red dots, right y-axis) is increasing, which is indicated by the dotted lines as an guide for the eye.

SUPPLEMENTARY REFERENCES

[1] Bernert, C. *et al.* Transient laser-induced breakdown of dielectrics in ultrarelativistic laser-solid interactions. *Physical Review Applied* **19**, 014070 (2023)
